# Re: ChatGPT encounters multiple opportunities and challenges in neurosurgery

**DOI:** 10.1097/JS9.0000000000001086

**Published:** 2024-01-18

**Authors:** Chengxing Qian, Yi Fang

**Affiliations:** a Department of Neurosurgery, Tongling People’s Hospital, Tongling; b Department of Neurosurgery, Peking Union Medical College Hospital, Chinese Academy of Medical Sciences and Peking Union Medical College, Beijing, People’s Republic of China

*Dear Editor*,

ChatGPT has become a highly acclaimed artificial intelligence (AI), generating text that closely resembles human conversation and providing users with natural and captivating responses, sparking widespread attention. Recently, I had the opportunity to explore an insightful article on the potential applications of ChatGPT in the field of neurosurgery^1^. This article delved into the capabilities and limitations of ChatGPT, particularly in the context of patient interactions, medical education, and clinical decision-making. As neurosurgery residents, the potential integration of ChatGPT into our daily workflow and patient care processes has prompted reflection on its practical implications for the future of our specialty.

The article underscores ChatGPT’s potential applications in preoperative planning, intraoperative management, and postoperative rehabilitation. The concept of leveraging an AI-powered virtual assistant for delivering extensive information to patients regarding their conditions, treatment alternatives, and postoperative care is captivating. Such a tool has the potential to empower patients, enabling them to make more informed decisions about their health and potentially enhancing overall satisfaction with their healthcare experiences. Moreover, in the realm of medical education, the article suggests that ChatGPT could serve as a valuable resource for neurosurgery students and residents. It could offer information on various topics related to neurosurgery, including anatomy, surgical techniques, and postoperative care. This interactive approach, supplemented by tools like quizzes and educational materials, could contribute to more effective and personalized learning experiences^2^. This holds great promise for regions with scarce neurosurgical resources, potentially shortening the learning curve for resident physicians. Additionally, the ChatGPT 4.0 version offers a hopeful solution for utilizing extensive clinical image data in education.

However, as with any technological advancement, it is crucial to approach the integration of ChatGPT with caution. The model’s inability to conduct physical examinations, interpret diagnostic imagery, and discern a patient’s emotional state raises valid concerns. Additionally, the potential for biased or erroneous responses, especially in critical areas like medical diagnosis, necessitates a thoughtful and cautious approach. Furthermore, there is a concern about the phenomenon of ChatGPT distorting facts, which could lead to patients developing doubts and misunderstandings about medical decisions made by neurosurgery^3^.

From a practical standpoint, even though ChatGPT presented near-passing performance on neurosurgery board examinations, the implementation of ChatGPT in neurosurgery should be viewed as a supplementary tool rather than a replacement for human expertise^4,5^. Our intricate field requires a nuanced understanding of individual patient cases, empathy, and the ability to navigate the subtleties of language and emotion, which current AI models may lack.

In conclusion, while ChatGPT holds promise in enhancing certain aspects of neurosurgical practice, it is imperative for us, as practitioners, to engage in a thoughtful evaluation of its benefits and limitations. As we tread into the future of neurosurgery, the collaboration between human expertise and AI assistance could potentially yield remarkable advancements in patient care, education, and clinical decision-making.

## Ethical approval

Not applicable.

## Consent

Not applicable.

## Sources of funding

Not applicable.

## Author contribution

All authors read and approved the final version of the manuscript.

## Conflicts of interest disclosure

There are no conflicts of interest.

## Research registration unique identifying number (UIN)

Not applicable.

## Guarantor

All the authors of this paper accept full responsibility for the work and/or the conduct of the study, have access to the data, and control the decision to publish.

## Data availability statement

No primary data were generated and reported in this manuscript. Therefore, data have not become available to any academic repository.
